# Filariasis of the breast, diagnosed by fine needle aspiration cytology

**DOI:** 10.4103/0256-4947.55178

**Published:** 2009

**Authors:** Naorem G. Singh, Leena Chatterjee

**Affiliations:** a From the Department of Pathology, Al Jahra Hospital, Jahra, Kuwait; b From the Super Religare Laboratory Limited, New Delhi, India

**To the Editor:** Bancroftian filariasis has a worldwide distribution, with disease prevalence in Africa, Asia including China, India and Southeast Asia, the Caribbean islands, Central and South America.^1^ It is a major health problem in tropical countries. The breast is an unusual site for the occurrence of a filarial nodule and only a few such cases have been documented.^2^–^5^ Microfilariae (MF) and adult worms are detected by needle aspirates from the breast, which aid in the diagnosis and treatment of the disease. Other rare unusual sites in which MF are reported include the thyroid nodule,^6^ salivary gland,^7^ cervicovaginal smear,^8^ ovarian cyst fluid,^8^ bronchial brushings,^8^ effusion fluid,^8^ and gastric brush.^9^

**Figure 1:**
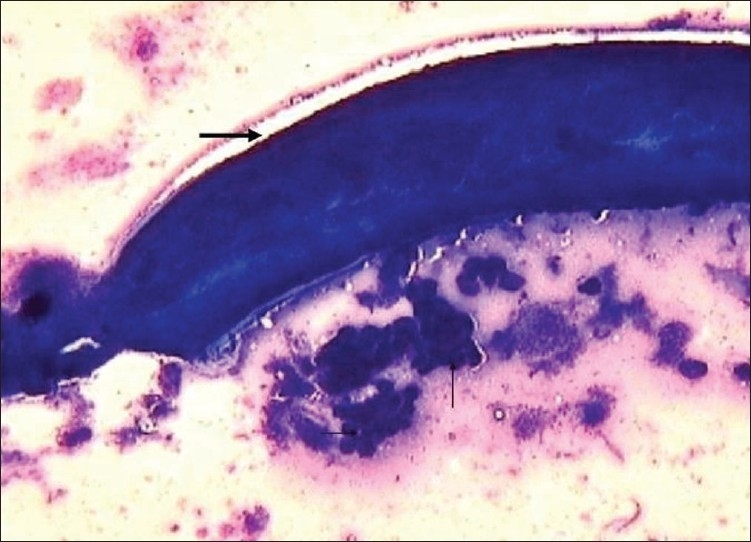
Aspirate showing female adult filarial worm (thick arrow) along with discharged numerous coiled form of larvae (thin arrow) outside the adult worm (May-Grunwald-giemsa stain ×100).

**Figure 2:**
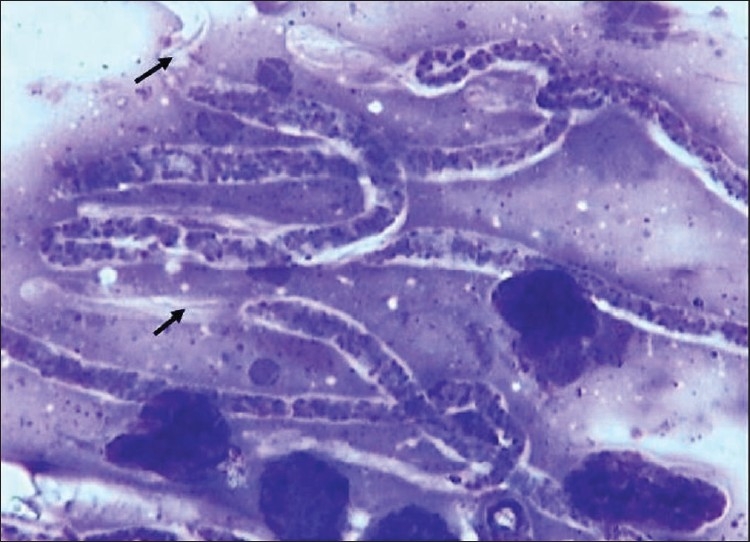
Microfilarial larvae showing sheathed, terminal nuclei and a caudal space (arrow) at the posterior end (May-Grunwald-giemsa stain ×400).

A 29-year-old female presented at the Super Religare Laboratory (formerly SRL Ranbaxy) Gurgaon, India, with a complaint of a mass in the right breast for 3 months duration. On palpation, a small subcutaneous nodule of approximately 9 mm in diameter was identified in the areola. The nodule was firm and nontender. The overlying skin was normal. There was no nipple discharge and the axillary lymph nodes were not palpable. Other physical and medical examinations were unremarkable. Fine needle aspiration (FNA) of the breast nodule was performed using a 23-gauge needle attached to a 10 mL disposable syringe. The aspirate was smeared on a slide, air-dried, and stained with May-Grunwald-giemsa stain. Cytologic examination revealed a gravid female adult worm along with numerous MF both in coiled and uncoiled forms (Figure 1) The MF were sheathed with elongated terminal nuclei and a caudal space at the posterior end (Figure 2) There were scattered inflammatory cells comprising polymorphs, lymphocytes and few histiocytes. No ductal cells were included in the aspirated material. A diagnosis of MF of the breast morphologically consistent with the *Wuchereria bancrofti* was entertained. Since our establishment was a diagnostic laboratory, the patient was subsequently referred to the concerned physician for the treatment and follow-up, and we have no further information on her disposition.

Bancroftian filariasis has a worldwide distribution. Insects, particularly mosquitoes serve as the intermediate host. While taking a blood meal, the insect ingests MF. Over 2 to 3 weeks, the MF develop within the insect into infective third-stage larvae. They reenter the definitive human host when the insect feeds again. The larvae mature into adult worm which lives for 10 to 15 years and produces MF. The patient usually presents with a solitary painless nodule in the upper outer quadrant of the breast. Central and periareolar regions are also involved with notable frequency as seen in our case. Multiple lesions are uncommon.^5^ Most of the lesions manifest as subcutaneous hard mass nodule with cutaneous attachment. More recently, in endemic areas, FNA has been employed to diagnose cases of breast involvement.^2^–^5^ In the present case, an FNA smear yielded a fragment of gravid female adult worm along with numerous MF. Cytologic diagnosis of filariasis by FNA from other sites, other than the breast such as the testis, epididymis, thyroid, lung, lymph node and skin, has been reported. A review of these cases by Kaya and colleagues revealed that the positivity for the MF in blood examinations in these patients was approximately 12%.^10^ Therefore, because of the low yield and stringent sampling requirement of a blood examination, FNA cytology appears to be a more convenient and effective diagnostic method in patient with mass lesions. Thus to conclude, demonstration and identification of the parasite in the smear played a significant role in the prompt recognition of the disease and institution of specific therapy.
